# Comparison of Adiponectin Levels in Anorexia Nervosa, Bulimia Nervosa, Binge-Eating Disorder, Obesity, Constitutional Thinness, and Healthy Controls: A Network Meta-Analysis

**DOI:** 10.3390/life13051181

**Published:** 2023-05-13

**Authors:** Umit Tural, Allison Sparpana, Elizabeth Sullivan, Dan V. Iosifescu

**Affiliations:** 1 Clinical Research Division, The Nathan S. Kline Institute for Psychiatric Research, Orangeburg, NY 10962, USA; 2 Psychiatry Department, New York University School of Medicine, New York, NY 10016, USA

**Keywords:** adiponectin, anorexia nervosa, bulimia nervosa, binge-eating disorder, obesity, constitutional thinness, network meta-analysis

## Abstract

Adiponectin is a protein hormone that is produced and secreted primarily by adipose tissue. The levels of adiponectin in those with eating disorders, obesity, and healthy controls have been extensively studied. However, the general picture of the differences in adiponectin levels across the mentioned conditions is still unclear and fragmented. In this study, we pooled previous studies and performed a network meta-analysis to gain a global picture of comparisons of adiponectin levels across eating disorders, obesity, constitutional thinness, and healthy controls. Electronic databases were searched for anorexia nervosa, avoidant restrictive food intake disorder, binge-eating disorder, bulimia nervosa, healthy controls, night eating syndrome, obesity, and constitutional thinness in studies where adiponectin levels were measured. A total of 4262 participants from 50 published studies were included in the network meta-analysis. Adiponectin levels were significantly higher in participants with anorexia nervosa than in healthy controls (Hedges’ g = 0.701, *p* < 0.001). However, adiponectin levels in constitutionally thin participants were not significantly different from those of healthy controls (Hedges’ g = 0.470, *p* = 0.187). Obesity and binge-eating disorder were associated with significantly lower adiponectin levels compared to those of healthy controls (Hedges’ g = −0.852, *p* < 0.001 and Hedges’ g = −0.756, *p* = 0.024, respectively). The disorders characterized by excessive increases or decreases in BMI were associated with significant changes in adiponectin levels. These results suggest that adiponectin may be an important marker of severely disequilibrated homeostasis, especially in fat, glucose, and bone metabolisms. Nonetheless, an increase in adiponectin may not simply be associated with a decrease in BMI, as constitutional thinness is not associated with a significant increase in adiponectin.

## 1. Introduction

Eating disorders such as anorexia nervosa (AN), bulimia nervosa (BN), and binge-eating disorder (BED) affect millions of people each year, as the current one-year prevalence rate is estimated to be 1.66%, and the rate for women is as high as 2.62% [1]. The Centers for Disease Control and Prevention (CDC) estimates that 41.9% of adults 20 and older in the United States are obese [2]. Despite the prevalence and associated health effects of both eating disorders and obesity, there is still much that is unknown about their biomarkers. A more comprehensive understanding of such biomarkers may improve the identification and treatment of eating disorders and of the physical complications associated with both eating disorders and obesity. Obesity is listed under “Other Conditions That May Be a Focus of Clinical Attention” in the DSM-5, but it is not included in the DSM-5 as a mental disorder. However, there are robust associations between obesity and mental disorders (e.g., binge-eating disorder, depressive and bipolar disorders, and schizophrenia) as also stated in the DSM-5.

One such biomarker that is relevant to the clinical worsening of eating disorders is adiponectin, an amino-acid protein secreted primarily by adipose tissue and found abundantly in serum and plasma circulation [3]. This protein is thought to be largely involved in metabolic, cardiovascular, and inflammatory processes and is closely related to body mass index (BMI), making it especially relevant in the study of eating disorders and their effects on health [4,5,6,7].

Elevated levels of adiponectin have been shown to increase insulin sensitivity [8,9,10], reduce atherosclerosis [5], and regulate inflammation [6]. Insufficient levels of circulating adiponectin are closely related to obesity [3], insulin resistance [7], type II diabetes [5], and cardiovascular disorders [11,12]. 

Several studies have established a negative correlation between adiponectin levels and BMI [3,7]. In a study of thin, normal weight, overweight, and obese adolescents, mean levels of adiponectin were significantly lower in both the overweight and obese groups and significantly higher in the thin group as compared to the normal weight group [13]. 

Adiponectin levels can also be affected by the presence of eating disorders, especially AN. Two meta-analyses have confirmed significantly increased levels of adiponectin in AN compared to healthy controls [14,15]. However, it is not clear whether constitutional thinness (C.Thin) is associated with increased adiponectin levels compared to those of healthy controls [16,17,18].

Obese patients have decreased levels of mRNA and serum levels of adiponectin compared to those of healthy controls (HCs) [18,19,20,21,22]. An inverse relationship between adiponectin serum levels and BMI has been reported in cross-sectional studies repeatedly [23,24,25,26]. Notably, there may be an even stronger inverse relationship between adiponectin serum levels and body total fat mass [27,28,29].

Studies of adiponectin levels in BN patients show inconsistent findings, with BN patients having either higher [30,31], the same [32], or lower adiponectin levels as compared to HCs [18]. These results mimic the breadth of presentation of this disorder, consisting of heterogenous binge or purge habits.

Previous work has compared adiponectin levels between several of the aforementioned groups, but no global analysis has yet been conducted comparing those of AN, BN, obesity, and C.Thin patients to those of HCs. Network meta-analysis is a statistical technique for comparing multiple effect sizes simultaneously in a single analysis by combining direct and indirect evidence within a network of comparative trials [33]. This network meta-analysis seeks to pool and rank the comparisons of adiponectin levels in all these populations by their magnitude of effect sizes to create a global picture of adiponectin across disorders and give researchers a better understanding of the role and place of adiponectin across eating disorders, obesity, and constitutional thinness. 

## 2. Methods

This research was conducted per the Preferred Reporting Items for Systematic Reviews and Meta-Analyses (PRISMA) guidelines [34]. 

### 2.1. Data Sources and Search Strategy

The PubMed, EMBASE, and PsycNET search engines were examined with the Boolean processors *for “anorexia nervosa [Title/Abstract] OR bulimia nervosa [Title/Abstract] OR obesity [Title/Abstract] OR binge-eating disorder [Title/Abstract] OR thin [Title/Abstract] OR lean [Title/Abstract] OR healthy controls [Title/Abstract] OR normal [Title/Abstract] OR night eating syndrome [Title/Abstract] OR avoidant restrictive food intake [Title/Abstract] AND adiponectin [Title/Abstract]”.* “Species” and “article type” filters of the search engines were activated to limit the results to “human” and “clinical trial” studies. The reference lists of the articles found were searched for additional reports.

### 2.2. Selection of Studies

**Figure 1:**
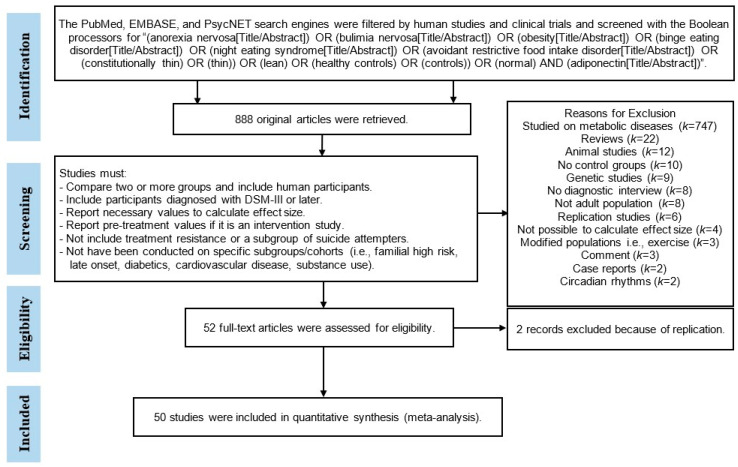
PRISMA flowchart of the article-selection process.

To reduce methodological heterogeneity, only studies with diagnoses based on the Diagnostic and Statistical Manual Version III (DSM-III) or subsequent versions (DSM-III-R, DSM-IV, DSM-IV-TR, or DSM-5) were included. Case reports, case series, and review articles were excluded. Studies reporting adiponectin levels in severe disorders (such as diabetes mellitus, kidney disease, polycystic ovary disease, cardiac disorders, systemic sclerosis, and fatty liver disease) were excluded from the meta-analysis. The PRISMA flowchart of the article selection process is presented in Figure 1. The PRISMA checklist for meta-analysis is included in Supplement Table S1. 

### 2.3. Inclusion and Exclusion Criteria

Studies including at least 2 (or more) study groups with participants who were either inpatients or outpatients, written in English, available from the earliest date to February 2023, were included in this meta-analysis. After a review of the abstracts of retrieved articles, full texts of the articles that included thin or obese participants or subjects diagnosed with AN or BN or BED compared to each other, or to a healthy control group, were evaluated. Only articles that reported the necessary information related to participants’ characteristics (especially N, SD, and mean of adiponectin level) or statistical test values to calculate effect sizes were included in this meta-analysis. If more than one subtype of AN (i.e., restrictive or bulimic/purgative) were included in a study, the subtypes’ means and standard deviations were pooled in a single group (3 studies, see Appendix A Table A1). The meta-analysis included only pre-intervention (baseline) values for studies that involved repeated measurements in two or more study groups. Single-arm studies designed as before-after interventions in patients or healthy controls were excluded because they did not compare two groups head to head or because it was not possible to generate effect size from unpaired comparison groups. 

### 2.4. Extraction of Data

A database file was prepared for the selected articles to record the first author’s name(s), year, sample size, sex, body mass index (BMI), mean age, assay method (the enzyme-linked immunosorbent assay-ELISA or radioimmunoassay-RIA), source of each metabolite (plasma or serum), and mean levels and SD of adiponectin. Medians and IRs, which were reported in two included studies, were converted to mean and SD approximations [35]. Consistent with the open science framework, the extracted dataset used in this meta-analysis can be found in Appendix A. 

### 2.5. Statistics and Meta-Analytic Strategy

A random network meta-analysis, with the residual (restricted) maximum likelihood (REML) algorithm to estimate tau2, combined direct and indirect evidence of bias-corrected effect sizes (Hedges’ g) from multiple-arm studies. The consistency of direct and indirect evidence extracted from each study was checked. Heterogeneity between studies was tested by Cochrane’s Q test, and the I2 value was inspected. Publication bias across the studies was explored with funnel plots and the Begg–Mazumdar and Egger tests. The SUCRA ranking score (Surface Under the Cumulative Ranking) was used to evaluate which diagnosis in the network was the most likely to be associated with the highest effect size in the network meta-analysis. All analyses were performed using the “network meta-analysis” [36] and “netmeta” [37] packages in the Stata (version 14.1) and R software (version 14.2), respectively [38,39].

## 3. Results

**Figure 2:**
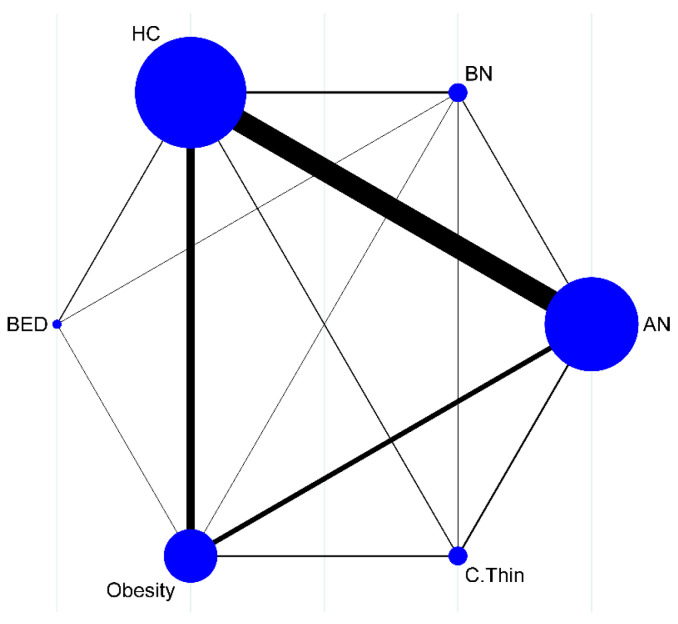
Successfully connected network map (circle size is associated with the number of studies, and line width is associated with the number of comparisons).

A pool of 4262 participants, 1265 AN (*k* = 39), 75 BN (*k* = 5), 57 BED (*k* = 3), 867 obese (*k* = 17), 426 C.Thin (*k* = 5), and 1572 healthy controls (*k* = 46), obtained from a total of 50 study articles, successfully generated a connected network (Figure 2). 

**Figure 3:**
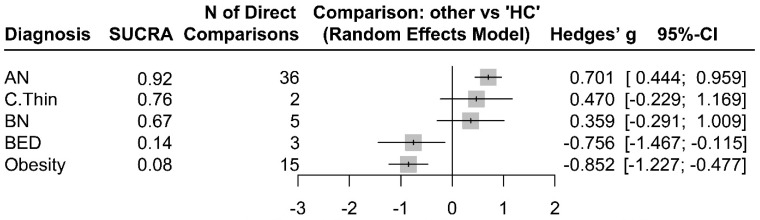
Magnitudes of standardized adiponectin differences (Hedges’ g) in AN, BED, BN, Obesity, and C.Thin compared to HC.

The electronic database search did not retrieve any results on night eating syndrome or avoidant restrictive food intake disorder, so they could not be included in the current network meta-analysis. Appendix A Table A1 presents the summarized study characteristics, design, and effect sizes. The Forest plot graph summarizes the pooled effect sizes in each diagnostic group vs. HCs that were extracted from the 50 studies (Figure 3). 

### 3.1. Comparisons of Study Groups vs. HCs

As seen in Figure 3, patients with AN had significantly increased adiponectin levels compared to those of HCs (Hedges’ g = 0.701, z = 5.34, *p* < 0.001). The adiponectin levels were also significantly decreased in patients with BED (Hedges’ g = −0.756, z = −2.25, *p* = 0.024) and obesity (Hedges’ g = −0.852, z = −4.45, *p* < 0.001) compared to those of HCs. Although constitutionally thin subjects (Hedges’ g = 0.4701, z = 1.32, *p* = 0.1873) and patients with BN (Hedges’ g = 0.359, z = 1.08, *p* = 0.279) had numerically increased levels of adiponectin compared to those of HCs, the differences were not statistically significant. There was significant within-design (Q = 456.98, df = 42, *p* < 0.001) and between-design (Q = 201.74, df = 18, *p* < 0.001) heterogeneity (Total *I*^2^ = 91%).

### 3.2. Consistency of Direct and Indirect Evidence

**Figure 4:**
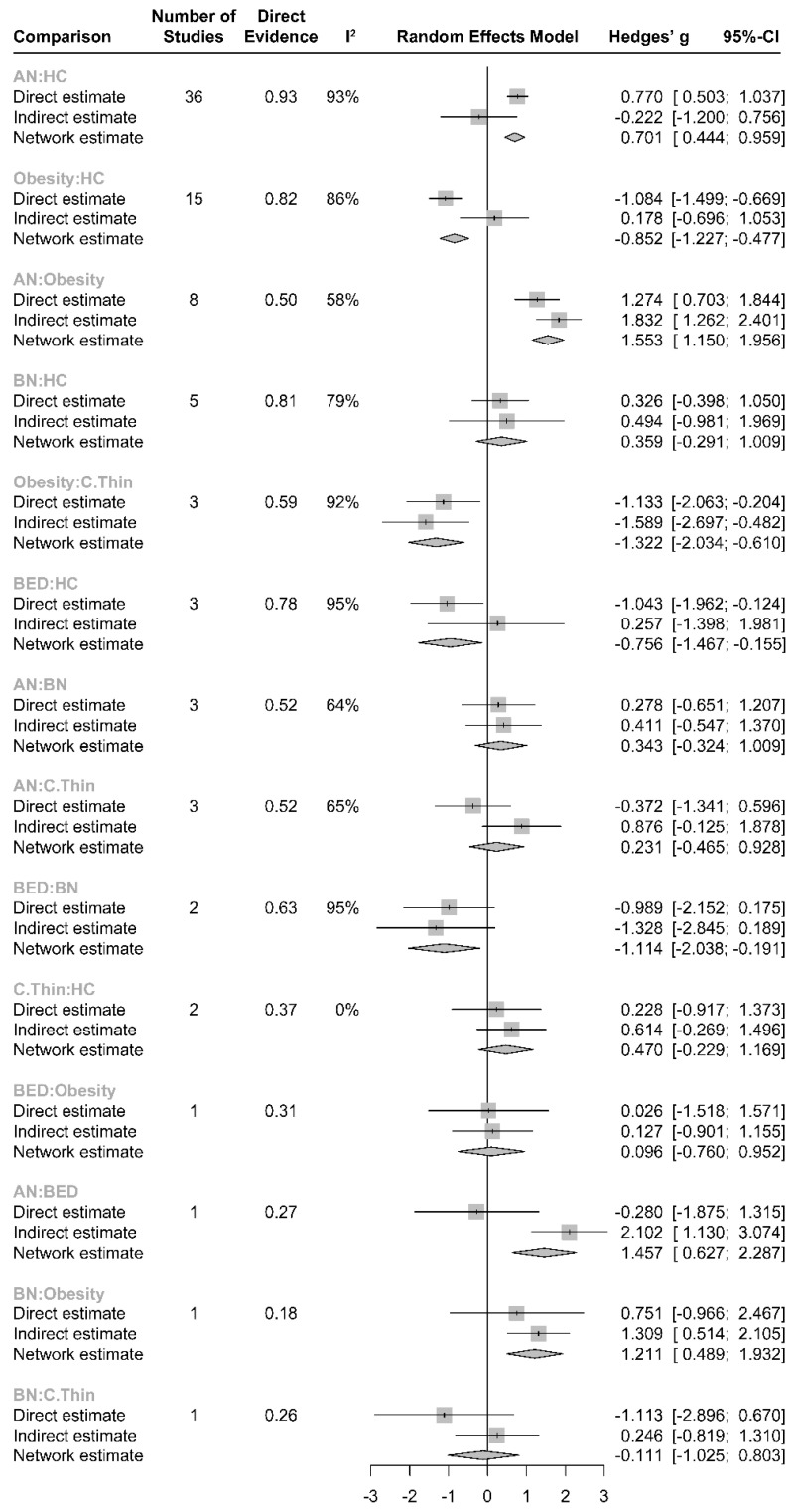
Split forest of direct evidence (%) vs. indirect evidence in effect size.

There was no overall inconsistency across the study arms between direct and indirect evidence (χ^2^_(18)_ = 28.22, *p* = 0.0588). Although global inconsistency was not significant (Supplement Table S2), individual comparisons of direct and indirect evidence obtained from obese vs. HC (diff = −1.26, z = −2.56, *p* = 0.011) and from AN vs. BED (diff = −2.38, z = 2.50, *p* = 0.012) comparisons yielded significant differences. A split forest of direct and indirect evidence is summarized in Figure 4. The net heat plot, which can be found in Supplement Figure S1, is a matrix visualization that highlights hot spots of inconsistent contribution of the corresponding design (diagonally) and inconsistency between direct and indirect evidence in a network estimate (off-diagonal).

### 3.3. Risk of Bias in Included Studies

Bias assessment of the included papers was performed according to the *Cochrane Handbook for Systematic Reviews of Interventions Version 5.1.0*. [40]. There was no noticeable evidence of bias in the studies included in the meta-analysis (Supplement Figure S2). More than 65% of the papers were assessed to have a low risk of bias.

### 3.4. Publication Bias and Small Study Effect

Adjusted funnel plot and publication bias statistics supported an unbiased distribution (Supplement Figure S3). There was no significant publication bias or small study effect in the meta-analysis (Begg–Mazumdar *p* = 0.304; Egger test *p* = 0.110, Thomson–Sharp *p* = 0.760). 

## 4. Discussion

We have compared the adiponectin levels across eating disorders, obesity, and constitutional thinness to those of healthy controls. The main finding of the meta-analysis was that adiponectin is significantly increased in AN patients compared to HCs; however, constitutionally thin people do not have significantly elevated adiponectin levels compared to those of HCs. This finding may suggest that the increases in adiponectin levels in AN cannot be explained simply by low BMI, as constitutional thinness is not associated with a significant increase in adiponectin compared to the levels in HCs. Thus, adiponectin increases in AN might be associated with heavily disequilibrated bodily physiological functions in many systems of the body rather than simply low BMI. Alternatively, adiponectin levels may be strongly associated with the clinical symptoms observed in AN such as amenorrhea and bone mineral loss, which again puts a significant stress on physiological systems [41,42,43]. These aforementioned symptoms are not observed in constitutionally thin people and may therefore constitute the line between health and disease. Previous studies have also shown significant relationships between various inflammatory factors and adiponectin [15,24,44]. The current findings further highlight the role of heavily disturbed general body functions in AN compared to constitutional thinness. Moreover, it is not yet clear whether adiponectin elevation in AN is permanent or if levels return to normal following re-feeding, since studies on re-feeding of AN patients have shown conflicting results [45,46]. Therefore, it can be hypothesized that the slightly increased adiponectin levels may play a protective role in maintaining energy levels during starvation, but sharply increased adiponectin levels may represent a compensatory mechanism during heavy disruption in metabolism as observed in AN. Furthermore, it can also be hypothesized that the increased adiponectin levels, which as noted may play a protective role in maintaining energy levels during starvation, are not needed in non-disordered weight loss. Rough calculations of means by groups can be found in Supplement Figure S4: the mean adiponectin levels in participants with AN are more than two times higher than in constitutionally thin people. 

Obesity, which is known to be associated with disequilibrated physiological functions, has significantly decreased adiponectin levels as compared to those of HCs. It is thought that adipokine drop in obesity is one of the key events in promoting systemic metabolic dysfunction, cardiovascular disease, and colorectal cancers [47]. BED appears to be associated with weight gain in the long term, though it can also be seen in non-obese persons [48,49]. We have found significantly lower levels of adiponectin in BED patients compared to HCs; however, it should be noted that the number of the studies in the BED arm was limited (*k* = 3) in the current network meta-analysis. Of note, the National Comorbidity Survey has reported that BED is associated with a lifetime of increased BMI (OR = 4.9, 95%CI = 2.2–11.0) compared to those without an eating disorder even after controlling for age and sex [49]. Therefore, lowered adiponectin levels are reasonable given the expected increase in BMI in BED. The same study reported that the majority of patients with BN have a normal BMI with no significant BMI changes across their lifetimes [49]. Additionally, DSM-5 classification does not seek BMI change in the diagnosis of BN. These findings support our result of nonsignificant changes in adiponectin levels in BN patients compared to HCs. Symptoms of severe destruction in bodily functions are not expected in BN; thus, significant changes in adiponectin levels may not accompany BN.

As expected, the limitations of the current meta-analysis are related to the limitations of the studies we pooled. The most important shortcomings were missing sample characteristics (sex, age, race, and socioeconomic status) and insufficient data on moderators, such as duration of the disorder, severity measurements, number of episodes, the magnitude and speed of weight loss, familial predisposition, or comorbidity, which may influence adiponectin levels. For example, the duration of AN and magnitude of weight loss could have also been significant moderators of adiponectin, but insufficient information was presented in the included studies for this analysis. Another limitation was the lack of a clear definition of constitutional thinness. In the future, more robust information about adiponectin will require studies in which those mentioned variables will be controlled. 

The presence of heterogeneity in the current network meta-analysis suggests that the variation in the study outcomes was significantly beyond expectations. Therefore, there may be other factors contributing to the extracted effect sizes from the studies other than the diagnosis. The aforementioned missing features of the pooled studies may have increased the heterogeneity. Single-arm studies (without comparisons) were excluded from the meta-analysis because it was not possible to extract a standardized mean difference between the groups. This exclusion procedure might have impacted the results. There was no publication bias in the pooled studies, which could contribute to reliable effect size estimations. Since the current network meta-analysis included a good number of studies, a low statistical power for detecting publication bias was not expected. There was no significant inconsistency between the direct and indirect evidence of the effect size. Therefore, our conclusions are valid even though only direct comparisons were considered.

However, the current network meta-analysis cannot clarify the cause of the increase or decrease in adiponectin levels in the studied populations. The current network analysis cannot tell us if BMI in healthy people sometimes drops to the levels of those of with AN. Nonetheless, these findings support that multiple physiological changes occur in AN, BED, and obesity, and significant changes in adiponectin may not be solely explained by BMI, as a similar change has not been found in constitutionally thin participants or in BN participants. Therefore, a significant change in adiponectin levels may represent heavily disturbed physiological equilibrium.

## Data Availability

Consistent with the open data policy, the full dataset used in this meta-analysis can be found in Appendix A.

## Supplementary Materials

The following supporting information can be downloaded at: https://www.mdpi.com/article/10.3390/life13051181/s1, Table S1: PRISMA checklist; Table S2: Comparisons of direct and indirect evidences; Figure S1: Netheat plot; Figure S2: Risk of bias assessments; Figure S3: Funnel plots for publication bias; Figure S4: Raw mean adiponectin levels by diagnosis.

## Appendix A

**Table A1:** General features of the studies included in the network meta-analysis.

Study	Group 1			Group 2			Design	Group 1	Group 2	Effect Size (Hedges’ g)	Standard Error
	Mean Adiponectin (µg/mL)	SD	N	Mean Adiponectin (µg/mL)	SD	N					
[50]	18.16	14.35	21	6.10	15.71	59	AN Obesity	AN	Obesity	0.777	0.262
[51]	6.06	3.93	9	7.59	1.83	9	AN HC	AN	HC	−0.475	0.480
[24]	7.52	4.09	12	9.32	3.42	11	AN HC Obesity	AN	HC	−0.538	0.423
[24]	7.52	4.09	12	4.90	1.72	11	AN HC Obesity	AN	Obesity	0.783	0.430
[24]	9.32	3.42	11	4.90	1.72	11	AN HC Obesity	HC	Obesity	1.320	0.460
[52]	0.58	0.10	30	0.49	0.05	30	HC Obesity	HC	Obesity	1.124	0.279
[53]	6.89	2.62	20	5.39	1.91	27	AN HC	AN	HC	0.659	0.303
[54]	6.70	3.25	23	5.19	1.56	43	AN HC	AN	HC	0.653	0.265
[55]	12.24	5.54	20	10.10	4.90	25	AN HC	AN	HC	0.406	0.303
[56]	16.10	4.59	26	11.80	4.41	24	AN HC	AN	HC	0.940	0.299
[57]	38.23	18.43	12	24.94	12.39	18	AN HC	AN	HC	0.859	0.391
[58]	57.80	26.01	17	33.00	28.00	17	AN HC Obesity	AN	HC	1.260	0.357
[58]	57.80	26.01	17	18.70	12.50	51	AN HC Obesity	AN	Obesity	1.987	0.321
[58]	33.00	28.00	17	18.70	12.50	51	AN HC Obesity	HC	Obesity	0.727	0.286
[59]	46.40	15.81	10	28.00	10.05	12	AN HC	AN	HC	1.365	0.484
[60]	36.50	17.85	17	19.90	9.44	17	AN HC	AN	HC	1.135	0.373
[61] *	33.99	14.89	41	20.58	8.10	41	AN HC	AN	HC	1.108	0.238
[62]	7.77	7.66	23	23.48	8.88	45	BED HC Obesity	BED	HC	−2.460	0.300
[62]	7.77	7.66	23	7.60	2.16	60	BED HC Obesity	BED	Obesity	0.026	0.245
[62]	23.48	8.88	45	7.60	2.16	60	BED HC Obesity	HC	Obesity	2.486	0.253
[13]	2.30	0.90	133	1.10	0.50	19	HC Obesity C.Thin	HC	Obesity	1.348	0.255
[13]	2.30	0.90	133	2.50	1.00	31	HC Obesity C.Thin	HC	C.Thin	−0.225	0.200
[13]	1.10	0.50	19	2.50	1.00	31	HC Obesity C.Thin	Obesity	C.Thin	−1.573	0.303
[44]	10.72	2.60	26	10.34	3.77	24	AN HC	AN	HC	0.116	0.283
[63]	36.50	18.87	19	19.90	9.16	16	AN HC	AN	HC	1.064	0.365
[64]	19.60	10.44	10	11.20	1.32	7	AN HC	AN	HC	0.981	0.528
[32] *	46.63	22.13	26	29.95	13.79	15	AN BN HC	AN	BN	0.927	0.338
[32]	46.63	22.13	26	26.01	8.52	12	AN BN HC	AN	HC	1.146	0.368
[32]	29.95	13.79	15	26.01	8.52	12	AN BN HC	BN	HC	0.219	0.388
[25]	26.21	4.18	16	15.81	3.55	16	HC Obesity	HC	Obesity	2.612	0.496
[65]	12.20	4.20	21	10.00	3.60	19	AN HC	AN	HC	0.549	0.323
[21]	23.12	8.92	20	16.67	4.89	28	AN HC Obesity	AN	HC	0.995	0.304
[21]	23.12	8.92	20	14.23	5.60	28	AN HC Obesity	AN	Obesity	1.372	0.315
[19]	16.67	4.89	28	14.23	5.60	28	AN HC Obesity	HC	Obesity	0.376	0.269
[66]	11.95	4.91	34	13.30	5.58	14	AN BED BN HC	AN	BED	−0.280	0.318
[66]	11.95	4.91	34	12.08	3.3	14	AN BED BN HC	AN	BN	−0.027	0.318
[66]	11.95	4.91	34	11.37	4.8	41	AN BED BN HC	AN	HC	0.120	0.232
[66]	13.3	5.58	14	12.08	3.3	14	AN BED BN HC	BED	BN	0.253	0.378
[66]	13.3	5.58	14	11.37	4.8	41	AN BED BN HC	BED	HC	0.311	0.400
[66]	12.08	3.3	14	11.37	4.8	41	AN BED BN HC	BN	HC	0.147	0.310
[67]	58.44	37.94	28	33.24	27.19	38	AN HC Obesity	AN	HC	1.087	0.257
[67]	58.44	37.94	28	17.02	10.44	77	AN HC Obesity	AN	Obesity	1.786	0.245
[67]	33.24	27.19	38	17.02	10.44	77	AN HC Obesity	HC	Obesity	0.699	0.203
[68] ^$^	11.77	5.74	17	8.1	3.36	50	HC Obesity	HC	Obesity	0.889	0.292
[20] ^$^	16.78	13.12	49	8.33	4.85	27	HC Obesity	HC	Obesity	0.764	0.248
[69]	19.00	7.70	50	12.00	5.80	50	AN HC	AN	HC	1.019	0.213
[20]	6.39	1.70	27	4.82	1.30	26	AN HC Obesity	AN	HC	0.983	0.286
[20]	6.39	1.70	27	4.30	1.70	27	AN HC Obesity	AN	Obesity	1.309	0.292
[21]	4.82	1.30	26	4.30	1.70	27	AN HC Obesity	HC	Obesity	0.326	0.276
[17]	33.50	25.50	9	37.20	13.28	10	AN C.Thin	AN	C.Thin	−0.176	0.461
[70]	12.80	5.27	23	12.50	7.79	21	AN HC	AN	HC	0.045	0.302
[71]	13.30	6.10	17	11.90	7.10	19	AN HC	AN	HC	0.206	0.335
[45]	16.80	3.56	20	11.90	3.30	13	AN HC	AN	HC	1.381	0.399
[72]	4.91	2.37	20	12.91	4.27	20	BED BN HC	BED	BN	−2.234	0.381
[72]	4.91	2.37	20	8.69	3.69	20	BED BN HC	BED	HC	−1.057	0.332
[72]	12.91	4.27	20	8.69	3.69	20	BED BN HC	BN	HC	1.178	0.335
[22]	13.70	6.30	11	9.56	2.98	11	AN HC Obesity	AN	HC	0.954	0.446
[22]	13.70	6.30	11	7.57	1.90	10	AN HC Obesity	AN	Obesity	1.412	0.477
[22]	9.56	2.98	11	7.57	1.90	10	AN HC Obesity	HC	Obesity	0.459	0.441
[73]	7.80	5.80	156	6.20	5.70	164	HC Obesity	HC	Obesity	0.278	0.112
[74] *	11.22	6.65	24	11.09	4.86	14	AN HC	AN	HC	0.021	0.336
[75]	16.43	5.85	15	11.09	6.31	20	AN HC	AN	HC	0.853	0.358
[41]	43.67	48.32	86	24.35	11.55	21	AN HC	AN	HC	0.438	0.245
[76]	30.50	15.80	11	22.60	10.90	26	AN HC	AN	HC	0.618	0.368
[77]	16.53	6.41	51	12.67	4.48	106	AN HC	AN	HC	0.741	0.176
[16]	16.10	5.40	220	17.50	5.00	360	Obesity C.Thin	Obesity	C.Thin	−0.271	0.086
[78] *	13.84	4.98	27	9.70	3.90	24	AN HC	AN	HC	0.905	0.296
[31]	13.32	3.71	15	10.58	2.87	45	BN HC	BN	HC	0.874	0.309
[18]	11.00	7.80	31	11.50	6.20	11	AN BN HC Obesity C.Thin	AN	BN	−0.065	0.351
[18]	11.00	7.80	31	18.30	9.80	16	AN BN HC Obesity C.Thin	AN	HC	−0.945	0.319
[18]	11.00	7.80	31	5.70	2.00	9	AN BN HC Obesity C.Thin	AN	Obesity	0.686	0.383
[18]	11.00	7.80	31	20.10	7.60	6	AN BN HC Obesity C.Thin	AN	C.Thin	−1.178	0.458
[18]	11.50	6.20	11	18.30	9.80	16	AN BN HC Obesity C.Thin	BN	HC	−0.880	0.399
[18]	11.50	6.20	11	5.70	2.00	9	AN BN HC Obesity C.Thin	BN	Obesity	0.751	0.454
[18]	11.50	6.20	11	20.10	7.60	6	AN BN HC Obesity C.Thin	BN	C.Thin	−1.113	0.517
[18]	18.30	9.80	16	5.70	2.00	9	AN BN HC Obesity C.Thin	HC	Obesity	1.631	0.440
[18]	18.30	9.80	16	20.10	7.60	6	AN BN HC Obesity C.Thin	HC	C.Thin	−0.233	0.479
[18]	5.70	2.00	9	20.10	7.60	6	AN BN HC Obesity C.Thin	Obesity	C.Thin	−1.864	0.551
[79]	29.30	8.37	28	16.50	5.30	33	AN HC	AN	HC	1.837	0.309
[46]	14.97	6.74	76	11.65	4.58	30	AN HC	AN	HC	0.530	0.219
[80]	27.36	48.68	20	14.20	14.84	30	AN HC	AN	HC	0.396	0.292
[81]	31.30	13.50	19	29.60	6.90	19	AN C.Thin	AN	C.Thin	0.155	0.325
[26]	16.70	2.70	8	12.10	1.80	9	HC Obesity	HC	Obesity	1.927	0.613
[82]	40.24	6.76	308	15.02	5.87	164	AN HC	AN	HC	3.895	0.160

* Restrictive and bulimic/purgative subtypes of AN were pooled into a single group. ^$^ Median and IR values were converted to means and SDs.

## References

[1] Deloitte Access Economics (2020). The Social and Economic Cost of Eating Disorders in the United States of America: A Report for the Strategic Training Initiative for the Prevention of Eating Disorders and the Academy for Eating Disorders.

[2] Stierman B., Joseph A., Margaret C., Chen T.-C., Davy O., Steven F., Cheryl F. (2021). NHSR 158. National Health and Nutrition Examination Survey 2017–March 2020 Pre-Pandemic Data Files-Development of Files and Prevalence Estimates for Selected Health Outcomes.

[3] Arita Y., Kihara S., Ouchi N., Takahashi M., Maeda K., Miyagawa J., Hotta K., Shimomura I., Nakamura T., Miyaoka K. (1999). Paradoxical Decrease of an Adipose-Specific Protein, Adiponectin, in Obesity. Biochem. Biophys. Res. Commun..

[4] Simpson K.A., Singh M.A.F. (2008). Effects of Exercise on Adiponectin: A Systematic Review. Obesity.

[5] Okamoto Y., Kihara S., Ouchi N., Nishida M., Arita Y., Kumada M., Ohashi K., Sakai N., Shimomura I., Kobayashi H. (2002). Adiponectin Reduces Atherosclerosis in Apolipoprotein E-Deficient Mice. Circulation.

[6] Ajuwon K.M., Spurlock M.E. (2005). Adiponectin Inhibits LPS-Induced NF-KappaB Activation and IL-6 Production and Increases PPARgamma2 Expression in Adipocytes. Am. J. Physiol. Regul. Integr. Comp. Physiol..

[7] Hotta K., Funahashi T., Arita Y., Takahashi M., Matsuda M., Okamoto Y., Iwahashi H., Kuriyama H., Ouchi N., Maeda K. (2000). Plasma Concentrations of a Novel, Adipose-Specific Protein, Adiponectin, in Type 2 Diabetic Patients. Arterioscler. Thromb. Vasc. Biol..

[8] Fasshauer M., Klein J., Kralisch S., Klier M., Lössner U., Blüher M., Paschke R. (2004). Growth Hormone Is a Positive Regulator of Adiponectin Receptor 2 in 3T3-L1 Adipocytes. FEBS Lett..

[9] Yamauchi T., Kamon J., Waki H., Terauchi Y., Kubota N., Hara K., Mori Y., Ide T., Murakami K., Tsuboyama-Kasaoka N. (2001). The Fat-Derived Hormone Adiponectin Reverses Insulin Resistance Associated with Both Lipoatrophy and Obesity. Nat. Med..

[10] Yamauchi T., Kamon J., Minokoshi Y., Ito Y., Waki H., Uchida S., Yamashita S., Noda M., Kita S., Ueki K. (2002). Adiponectin Stimulates Glucose Utilization and Fatty-Acid Oxidation by Activating AMP-Activated Protein Kinase. Nat. Med..

[11] Ouchi N., Kihara S., Arita Y., Maeda K., Kuriyama H., Okamoto Y., Hotta K., Nishida M., Takahashi M., Nakamura T. (1999). Novel Modulator for Endothelial Adhesion Molecules: Adipocyte-Derived Plasma Protein Adiponectin. Circulation.

[12] Marso S.P., Mehta S.K., Frutkin A., House J.A., McCrary J.R., Kulkarni K.R. (2008). Low Adiponectin Levels Are Associated with Atherogenic Dyslipidemia and Lipid-Rich Plaque in Nondiabetic Coronary Arteries. Diabetes Care.

[13] Ghomari-Boukhatem H., Bouchouicha A., Mekki K., Chenni K., Belhadj M., Bouchenak M. (2017). Blood Pressure, Dyslipidemia and Inflammatory Factors Are Related to Body Mass Index in Scholar Adolescents. Arch. Med. Sci..

[14] Karageorgiou V., Furukawa T.A., Tsigkaropoulou E., Karavia A., Gournellis R., Soureti A., Bellos I., Douzenis A., Michopoulos I. (2020). Adipokines in Anorexia Nervosa: A Systematic Review and Meta-Analysis. Psychoneuroendocrinology.

[15] Tural U., Iosifescu D.V. (2022). Adiponectin in Anorexia Nervosa and Its Modifiers: A Meta-regression Study. Int. J. Eat. Disord..

[16] Rohde K., Keller M., Horstmann A., Liu X., Eichelmann F., Stumvoll M., Villringer A., Kovacs P., Tönjes A., Böttcher Y. (2015). Role of Genetic Variants in ADIPOQ in Human Eating Behavior. Genes Nutr..

[17] Miljic D., Djurovic M., Pekic S., Doknic M., Stojanovic M., Milic N., Casanueva F.F., Ghatei M., Popovic V. (2007). Glucose Metabolism during Ghrelin Infusion in Patients with Anorexia Nervosa. J. Endocrinol. Investig..

[18] Tagami T., Satoh N., Usui T., Yamada K., Shimatsu A., Kuzuya H. (2004). Adiponectin in Anorexia Nervosa and Bulimia Nervosa. J. Clin. Endocrinol. Metab..

[19] Karczewska-Kupczewska M., Kowalska I., Nikolajuk A., Adamska A., Otziomek E., Gorska M., Straczkowski M. (2012). Hyperinsulinemia Acutely Increases Serum Macrophage Inhibitory Cytokine-1 Concentration in Anorexia Nervosa and Obesity. Clin. Endocrinol..

[20] Lejawa M., Osadnik K., Czuba Z., Osadnik T., Pawlas N. (2021). Association of Metabolically Healthy and Unhealthy Obesity Phenotype with Markers Related to Obesity, Diabetes among Young, Healthy Adult Men. Analysis of MAGNETIC Study. Life.

[21] Mariani S., Di Giorgio M.R., Rossi E., Tozzi R., Contini S., Bauleo L., Cipriani F., Toscano R., Basciani S., Barbaro G. (2020). Blood SIRT1 Shows a Coherent Association with Leptin and Adiponectin in Relation to the Degree and Distribution of Adiposity: A Study in Obesity, Normal Weight and Anorexia Nervosa. Nutrients.

[22] Nakahara T., Harada T., Yasuhara D., Shimada N., Amitani H., Sakoguchi T., Kamiji M.M., Asakawa A., Inui A. (2008). Plasma Obestatin Concentrations Are Negatively Correlated with Body Mass Index, Insulin Resistance Index, and Plasma Leptin Concentrations in Obesity and An-orexia Nervosa. Biol. Psychiatry.

[23] Kern P.A., Di Gregorio G.B., Lu T., Rassouli N., Ranganathan G. (2003). Adiponectin Expression from Human Adipose Tissue: Relation to Obesity, Insulin Resistance, and Tumor Necrosis Factor-Alpha Expression. Diabetes.

[24] Amitani M., Asakawa A., Amitani H., Kaimoto K., Sameshima N., Koyama K.I., Haruta I., Tsai M., Nakahara T., Ushikai M. (2013). Plasma Klotho Levels Decrease in Both Anorexia Nervosa and Obesity. Nutrition.

[25] Huang R., Song C., Li T., Yu C., Yao T., Gao H., Cao S., Yi X., Chang B. (2022). A Cross-Sectional Comparative Study on the Effects of Body Mass Index and Exercise/Sedentary on Serum Asprosin in Male College Students. PLoS ONE.

[26] Yu J.G., Javorschi S., Hevener A.L., Kruszynska Y.T., Norman R.A., Sinha M., Olefsky J.M. (2002). The Effect of Thiazolidinediones on Plasma Adiponectin Levels in Normal, Obese, and Type 2 Diabetic Subjects. Diabetes.

[27] Sirbu A.E., Buburuzan L., Kevorkian S., Martin S., Barbu C., Copaescu C., Smeu B., Fica S. (2018). Adiponectin Expression in Visceral Adiposity Is an Important Determinant of Insulin Resistance in Morbid Obesity. Endokrynol. Pol..

[28] Bellissimo M.P., Hsu E., Hao L., Easley K., Martin G.S., Ziegler T.R., Alvarez J.A. (2021). Relationships between Plasma Apelin and Adiponectin with Normal Weight Obesity, Body Composition, and Cardiorespiratory Fitness in Working Adults. J. Clin. Transl. Endocrinol..

[29] Manigrasso M.R., Ferroni P., Santilli F., Taraborelli T., Guagnano M.T., Michetti N., Davì G. (2005). Association between Circulating Adiponectin and Interleukin-10 Levels in Android Obesity: Effects of Weight Loss. J. Clin. Endocrinol. Metab..

[30] Monteleone A.M., Monteleone P., Marciello F., Pellegrino F., Castellini G., Maj M. (2017). Differences in Cortisol Awakening Response between Binge-Purging and Restrictive Patients with Anorexia Nervosa. Eur. Eat. Disord. Rev..

[31] Syk M., Ramklint M., Fredriksson R., Ekselius L., Cunningham J.L. (2017). Elevated Total Plasma-Adiponectin Is Stable over Time in Young Women with Bulimia Nervosa. Eur. Psychiatry.

[32] Housova J., Anderlova K., Krizová J., Haluzikova D., Kremen J., Kumstyrová T., Papezová H., Haluzik M. (2005). Serum Adiponectin and Resistin Concentrations in Patients with Restrictive and Binge/Purge Form of Anorexia Nervosa and Bulimia Nervosa. J. Clin. Endocrinol. Metab..

[33] Rouse B., Chaimani A., Li T. (2017). Network Meta-Analysis: An Introduction for Clinicians. Intern. Emerg. Med..

[34] Moher D., Liberati A., Tetzlaff J., Altman D.G. (2009). Preferred Reporting Items for Systematic Reviews and Meta-Analyses: The PRISMA Statement. J. Clin. Epidemiol..

[35] Wan X., Wang W., Liu J., Tong T. (2014). Estimating the Sample Mean and Standard Deviation from the Sample Size, Median, Range and/or Interquartile Range. BMC Med. Res. Methodol..

[36] White I.R. (2015). Network Meta-Analysis. Stata J. Promot. Commun. Stat. Stata.

[37] Balduzzi S., Rücker G., Nikolakopoulou A., Papakonstantinou T., Salanti G., Efthimiou O., Schwarzer G. (2023). netmeta: An R Package for Network Meta-Analysis Using Frequentist Methods. J. Stat. Softw..

[38] StataCorp (2015). Stata Statistical Software: Release 14.

[39] R Core Team (2021). R: A Language and Environment for Statistical Computing.

[40] Higgins J.P., Green S., Higgins J.P.T., Green S. (2011). Assessing Risk of Bias in Included Studies.

[41] Ostrowska Z., Ziora K., Oswiecimska J., Swietochowska E., Marek B., Kajdaniuk D., Wołkowska-Pokrywa K., Kos-Kudła B. (2014). Bone Metabolism, Osteoprotegerin, Receptor Activator of Nuclear Factor-ΚB Ligand and Selected Adipose Tissue Hormones in Girls with Anorexia Nervosa. Endokrynol. Pol..

[42] Wu N., Wang Q.-P., Li H., Wu X.-P., Sun Z.-Q., Luo X.-H. (2010). Relationships between Serum Adiponectin, Leptin Concentrations and Bone Mineral Density, and Bone Biochemical Markers in Chinese Women. Clin. Chim. Acta.

[43] Mathew H., Castracane V.D., Mantzoros C. (2018). Adipose Tissue and Reproductive Health. Metabolism.

[44] Guo L.J., Jiang T.J., Liao L., Liu H., He H.B. (2013). Relationship between Serumomentin-1 Level and Bonemineral Density in Girls with Anorexia Nervosa. J. Endocrinol. Investig..

[45] Modan-Moses D., Stein D., Pariente C., Yaroslavsky A., Ram A., Faigin M., Loewenthal R., Yissachar E., Hemi R., Kanety H. (2007). Modulation of Adiponectin and Leptin during Refeeding of Female Anorexia Nervosa Patients. J. Clin. Endocrinol. Metab..

[46] Tyszkiewicz-Nwafor M., Slopien A., Dmitrzak-Węglarz M., Rybakowski F. (2019). Adiponectin and Resistin in Acutely Ill and Weight-Recovered Adolescent Anorexia Nervosa: Association with Psychiatric Symptoms. World J. Biol. Psychiatry.

[47] Nakamura K., Fuster J.J., Walsh K. (2014). Adipokines: A Link between Obesity and Cardiovascular Disease. J. Cardiol..

[48] McCuen-Wurst C., Ruggieri M., Allison K.C. (2018). Disordered Eating and Obesity: Associations between Binge-Eating Disorder, Night-Eating Syndrome, and Weight-Related Comorbidities. Ann. N. Y. Acad. Sci..

[49] Hudson J.I., Hiripi E., Pope H.G.J., Kessler R.C. (2007). The Prevalence and Correlates of Eating Disorders in the National Comorbidity Survey Replication. Biol. Psychiatry.

[50] Alberti L., Gilardini L., Girola A., Moro M., Cavagnini F., Invitti C. (2007). Adiponectin Receptors Gene Expression in Lymphocytes of Obese and Anorexic Patients. Diabetes Obes. Metab..

[51] Amitani H., Asakawa A., Ogiso K., Nakahara T., Ushikai M., Haruta I., Koyama K., Amitani M., Cheng K., Inui A. (2013). The Role of Adiponectin Multimers in Anorexia Nervosa. Nutrition.

[52] Bahceci M., Gokalp D., Bahceci S., Tuzcu A., Atmaca S., Arikan S. (2007). The Correlation between Adiposity and Adiponectin, Tumor Necrosis Factor Alpha, Interleukin-6 and High Sensitivity C-Reactive Protein Levels. Is Adipocyte Size Associated with Inflammation in Adults?. J. Endocrinol. Investig..

[53] Baranowska-Bik A., Baranowska B., Martyńska L., Litwiniuk A., Kalisz M., Kochanowski J., Bik W. (2017). Adipokine Profile in Patients with Anorexia Nervosa. Endokrynol. Pol..

[54] Bosy-Westphal A., Brabant G., Haas V., Onur S., Paul T., Nutzinger D., Klein H., Hauer M., Müller M.J. (2005). Determinants of Plasma Adiponectin Levels in Patients with Anorexia Nervosa Examined before and after Weight Gain. Eur. J. Nutr..

[55] Buckert M., Stroe-Kunold E., Friederich H.-C., Wesche D., Walter C., Kopf S., Simon J.J., Herzog W., Wild B. (2017). Time Course of Adiponectin and Its Relationship to Psychological Aspects in Patients with Anorexia Nervosa during Inpatient Treatment. PLoS ONE.

[56] Delporte M.L., Brichard S.M., Hermans M.P., Beguin C., Lambert M. (2003). Hyperadiponectinaemia in Anorexia Nervosa. Clin. Endocrinol..

[57] Dolezalova R., Lacinova Z., Dolinkova M., Kleiblova P., Haluzikova D., Housa D., Papezova H., Haluzik M. (2007). Changes of Endocrine Function of Adipose Tissue in Anorexia Nervosa: Comparison of Circulating Levels versus Subcutaneous MRNA Expression. Clin. Endocrinol..

[58] Dolinkova M., Krizova J., Lacinova Z., Dolezalova R., Housova J., Krajickova J., Bosanska L., Papezova H., Haluzik M. (2006). [Polymorphisms of adiponectin and resistin genes in patients with obesity and anorexia nervosa]. Cas. Lek. Cesk..

[59] Dostálová I., Smitka K., Papežová H., Kvasničková H., Nedvídková J. (2007). Increased Insulin Sensitivity in Patients with Anorexia Nervosa: The Role of Adipocytokines. Physiol. Res..

[60] Dostalova I., Kavalkova P., Haluzakova D., Lacinova Z., Mraz M., Papezova H., Haluziik M. (2008). Plasma Concentrations of Fibroblast Growth Factors 19 and 21 in Patients with Anorexia Nervosa. J. Clin. Endocrinol. Metab..

[61] Elegido A., Gheorghe A., Sepúlveda A.R., Andrés P., Díaz-Prieto L.E., Graell M., Marcos A., Nova E. (2019). Adipokines, Cortisol and Cytokine Alterations in Recent Onset Anorexia Nervosa. A Case–Control Study. Endocrinol. Diabetes y Nutr..

[62] Erbaş İ.M., Paketçi A., Turan S., Şişman A.R., Demir K., Böber E., Abacı A. (2022). Low C1q Complement/TNF-Related Protein-13 Levels Are Related with Childhood Obesity but Not Binge Eating Disorder. J. Clin. Res. Pediatr. Endocrinol..

[63] Haluzikova D., Dostalova I., Kavalkova P., Roubicek T., Mraz M., Papezova H., Haluzik M. (2009). Serum Concentrations of Adipocyte Fatty Acid Binding Protein in Patients with Anorexia Nervosa. Physiol. Res..

[64] Heilbronn L.K., Milner K.-L., Kriketos A., Russell J., Campbell L.V. (2007). Metabolic Dysfunction in Anorexia Nervosa. Obes. Res. Clin. Pract..

[65] Iwahashi H., Funahashi T., Kurokawa N., Sayama K., Fukuda E., Okita K., Imagawa A., Yamagata K., Shimomura I., Miyagawa J.I. (2003). Plasma Adiponectin Levels in Women with Anorexia Nervosa. Horm. Metab. Res..

[66] Garella R., Cassioli E., Chellini F., Tani A., Rossi E., Idrizaj E., Guasti D., Comeglio P., Palmieri F., Parigi M. (2023). Defining the Molecular Mechanisms of the Relaxant Action of Adiponectin on Murine Gastric Fundus Smooth Muscle: Potential Translational Perspectives on Eating Disorder Management. Int. J. Mol. Sci..

[67] Krizova J., Dolinkova M., Lacinova Z., Sulek S., Dolezalova R., Housova J., Krajickova J., Haluzikova D., Bosanska L., Papezova H. (2008). Adiponectin and Resistin Gene Polymorphisms in Patients with Anorexia Nervosa and Obesity and Its Influence on Metabolic Phenotype. Physiol. Res..

[68] Larsen M.A., Isaksen V.T., Paulssen E.J., Goll R., Florholmen J.R. (2019). Postprandial Leptin and Adiponectin in Response to Sugar and Fat in Obese and Normal Weight Individuals. Endocrine.

[69] Maimoun L., Guillaume S., Lefebvre P., Philibert P., Bertet H., Picot M., Gaspari L., Paris F., Seneque M., Dupuys A. (2016). Evidence of a Link between Resting Energy Expenditure and Bone Remodelling, Glucose Homeostasis and Adipokine Variations in Adolescent Girls with Anorexia Nervosa. Osteoporos. Int..

[70] Misra M., Miller K., Almazan C., Ramaswamy K., Aggarwal A., Herzog D., Neubauer G., Breu J., Klibanski A. (2004). Hormonal and Body Composition Predictors of Soluble Leptin Receptor, Leptin, and Free Leptin Index in Adolescent Girls with Anorexia Nervosa and Controls and Relation to Insulin Sensitivity. J. Clin. Endocrinol. Metab..

[71] Misra M., Miller K., Cord J., Prabhakaran R., Herzog D., Goldstein M., Katzman D., Klibanski A. (2007). Relationships between Serum Adipokines, Insulin Levels, and Bone Density in Girls with Anorexia Nervosa. J. Clin. Endocrinol. Metab..

[72] Monteleone P., Fabrazzo M., Martiadis V., Fuschino A., Serritella C., Milici N., Maj M. (2003). Opposite Changes in Circulating Adiponectin in Women with Bulimia Nervosa or Binge Eating Disorder. J. Clin. Endocrinol. Metab..

[73] Nezhadali M., Mesbah-Namin S.A., Hedayati M., Akbarzadeh M., Najd Hassan Bonab L., Daneshpour M.S. (2022). Serum Adiponectin and Cortisol Levels Are Not Affected by Studied ADIPOQ Gene Variants: Tehran Lipid and Glucose Study. BMC Endocr. Disord..

[74] Nogueira J.-P., Maraninchi M., Lorec A.-M., Corroller A.B.-L., Nicolay A., Gaudart J., Portugal H., Barone R., Vialettes B., Valéro R. (2010). Specific Adipocytokines Profiles in Patients with Hyperactive and/or Binge/Purge Form of Anorexia Nervosa. Eur. J. Clin. Nutr..

[75] Omodei D., Pucino V., Labruna G., Procaccini C., Galgani M., Perna F., Pirozzi D., De Caprio C., Marone G., Fontana L. (2015). Immune-Metabolic Profiling of Anorexic Patients Reveals an Anti-Oxidant and Anti-Inflammatory Phenotype. Metabolism.

[76] Pannacciulli N., Vettor R., Milan G., Granzotto M., Catucci A., Federspil G., De Giacomo P., Giorgino R., DePergola G. (2003). Anorexia Nervosa Is Characterized by Increased Adiponectin Plasma Levels and Reduced Nonoxidative Glucose Metabolism. J. Clin. Endocrinol. Metab..

[77] Paslakis G., Aguera Z., Granero R., Sanchez I., Riesco N., Jimenez-Murcia S., Fernandez-Garcia J.C., Garrido-Sanchez L., Tinahones F.J., Casanueva F.F. (2019). Associations between Neuropsychological Performance and Appetite-Regulating Hormones in Anorexia Nervosa and Healthy Controls: Ghrelin’s Putative Role as a Mediator of Decision-Making. Mol. Cell. Endocrinol..

[78] Stoving R.K., Chen J.W., Glintborg D., Brixen K., Flyvbjerg A., Horder K., Frystyk J. (2007). Bioactive Insulin-like Growth Factor (IGF) I and IGF-Binding Protein-1 in Anorexia Nervosa. J. Clin. Endocrinol. Metab..

[79] Terra X., Auguet T., Aguera Z., Quesada I.M., Orellana-Gavalda J.M., Aguilar C., Jimenez-Murcia S., Berlanga A., Guiu-Jurado E., Menchon J.M. (2013). Adipocytokine Levels in Women with Anorexia Nervosa. Relationship with Weight Restoration and Disease Duration. Int. J. Eat. Disord..

[80] Tyszkiewicz-Nwafor M., Jowik K., Paszynska E., Dutkiewicz A., Słopien A., Dmitrzak-Weglarz M. (2022). Expression of Immune-Related Proteins and Their Association with Neuropeptides in Adolescent Patients with Anorexia Nervosa. Neuropeptides.

[81] Uzum A.K., Yucel B., Omer B., Issever H., Ozbey N.C. (2009). Leptin Concentration Indexed to Fat Mass Is Increased in Untreated Anorexia Nervosa (AN) Patients. Clin. Endocrinol..

[82] Ziora-Jakutowicz K.N., Zimowski J., Ziora K., Bednarska-Makaruk M., Świętochowska E., Gorczyca P., Szczepańska M., Machura E., Stojewska M., Gołąb-Jenerał K. (2021). Evaluation of the Frequency of ADIPOQ c.45 T>G and ADIPOQ c.276 G>T Polymorphisms in Adiponectin Coding Gene in Girls with Anorexia Nervosa. Endokrynol. Pol..

