# Supplementary material for: Comparison of Adiponectin Levels in Anorexia Nervosa, Bulimia Nervosa, Binge-Eating Disorder, Obesity, Constitutional Thinness, and Healthy Controls: A Network Meta-Analysis
Source: Life (Basel). 2023 May 13;13(5):1181. doi: 10.3390/life13051181 (PMC10220738; doi:10.3390/life13051181)
Supplement: Supplementary file 1 [file life-13-01181-s001.zip › life-2289163-supplementary.pdf]

Supplementary Materials for Comparison of Adiponectin Levels in Anorexia Nervosa, Bulimia Nervosa, Binge-Eating Disorder, Obesity, Constitutional Thinness, and Healthy Controls: A Network Meta-Analysis

Table S1. PRISMA Checklist

Section/topic	#	Checklist item	Reported on page #
**TITLE**			
Title	1	Identify the report as a systematic review, meta-analysis, or both.<br>*-Comparison of adiponectin levels in anorexia nervosa, bulimia nervosa, binge eating disorder, obesity, constitutional thinness, and healthy controls: A network meta-analysis*	1
**ABSTRACT**			
Structured summary	2	Provide a structured summary including, as applicable: background; objectives; data sources; study eligibility criteria, participants, and interventions; study appraisal and synthesis methods; results; limitations; conclusions and implications of key findings; systematic review registration number.<br>*-In the abstract*	2
**INTRODUCTION**			
Rationale	3	*Describe the rationale for the review in the context of what is already known.*<br>*-Adiponectin is a protein hormone which is produced and secreted primarily by adipose tissue. The levels of adiponectin in those with eating disorders, obesity, and healthy controls have been extensively studied. However, the general picture of the differences in adiponectin levels across the mentioned conditions is still unclear and fragmented.*	3-4
Objectives	4	Provide an explicit statement of questions being addressed with reference to participants, interventions, comparisons, outcomes, and study design (PICOS).<br>***Participants:*** *Controlled studies conducted with participants aged between 18 and 65 who were either inpatients or outpatients and written in English that available from the earliest date to July 2022 were included in this network meta-analysis. All studies comparing patients with AN, BN, BED, Obesity, constitutionally thins to controls. 1) the subjects had been diagnosed with diagnostic interview for AN, BN, BED and BMI for obesity constitutionally thinness 2) the study was*	5-6

		controlled and had included cases and controls, 3) the study had reported the means and standard derivations in both case and control groups, or detailed statistical test values to calculate effect size (SMD), or the authors had collected and shared with us by email the necessary data.<br>***Interventions****: No intervention. If study used an intervention on participants only the baseline (before the intervention) values were used.*<br>***Comparisons****: All groups compared with each other and effect sizes calculated from direct and indirect comparisons.*<br>***Outcome****: Effect sizes (hedges' g) of adiponectin differences.*<br>***Study Design****: Randomized controlled trials, cross-sectional studies, Cohort studies, observational studies.*	
**METHODS**			
Protocol and registration	5	Indicate if a review protocol exists, if and where it can be accessed (e.g., Web address), and, if available, provide registration information including registration number:<br>*-No, there is no review protocol published.*	
Eligibility criteria	6	Specify study characteristics (e.g., PICOS, length of follow-up) and report characteristics (e.g., years considered, language, publication status) used as criteria for eligibility, giving rationale.<br>*-Languages English, published data, all lengths of follow up, years since inception of database –today. See PICOS in objectives (point 4)*	6
Information sources	7	Describe all information sources (e.g., databases with dates of coverage, contact with study authors to identify additional studies) in the search and date last searched.<br>*-Pubmed (Medline), EMBASE and PsycNET inception till today*	5
Search	8	Present full electronic search strategy for at least one database, including any limits used, such that it could be repeated.<br>*-Using keywords "anorexia nervosa [Title/Abstract] OR (bulimia nervosa [Title/Abstract] OR (obesity [Title/Abstract] OR (binge eating disorder [Title/Abstract] OR (thin [Title/Abstract] OR (lean [Title/Abstract] (healthy controls [Title/Abstract] OR (normal [Title/Abstract] OR (night eating syndrome [Title/Abstract] OR (avoidant restrictive food intake [Title/Abstract] AND (adiponectin [Title/Abstract]"*. "Species" and "article type" filters of the search engines were activated to limit the results to "human" and "clinical trial" studies.	See manuscript, page 5
Study selection	9	State the process for selecting studies (i.e., screening, eligibility, included in systematic review, and, if applicable, included in the meta-analysis).<br>*- initial search in databases (available from the earliest date to February 2023)*	6 and see Figure 1

		- limit to English language<br>*- limit to human trials*<br>*- export search results into Mendeley under exclusion of duplicates, manually search for further duplicates*<br>*- make selection based on title and abstract, aided by Mendeley*<br>*- review selected full publications for eligibility,*<br>*- list reasons for exclusion and inclusion*<br>*- review references of published meta-analysis for possibly missing reports*	(PRISMA flow chart)
Data collection process	10	Describe method of data extraction from reports (e.g., piloted forms, independently, in duplicate) and any processes for obtaining and confirming data from investigators.<br>*-Extracted data: Raw means and SDs were extracted from the included studies for each of the study groups on an excel sheet. Relevant data such as diagnosis, comorbidities, clinical assessment scores, sex ratios, mean age, duration of illness and BMI also were extracted.*	6
Data items	11	List and define all variables for which data were sought (e.g., PICOS, funding sources) and any assumptions and simplifications made.	
Risk of bias in individual studies	12	Describe methods used for assessing risk of bias of individual studies (including specification of whether this was done at the study or outcome level), and how this information is to be used in any data synthesis.<br>*-Performed fully based on Cochrane Handbook for Systematic Reviews of Interventions version 5.1.0. The vast majority of included studies are qualified as low risk of bias. And, graphical presentation can be found as a supplemental file.*	6
Summary measures	13	State the principal summary measures (e.g., risk ratio, difference in means).<br>-Network meta-analysis:<br>*1. Standardized mean differences in adiponectin levels amongst the groups (hedges' g)*<br>*2. SUCRA probability ranking*	7
Synthesis of results	14	Describe the methods of handling data and combining results of studies, if done, including measures of consistency (e.g., I^2) for each meta-analysis.<br>*For each analysis, effect size (Hedges' g), pooled random effect size and I^2 calculated. Sensitivity analyses and publication bias were assessed.*	7

Section/topic	#	Checklist item	Reported on page #
Risk of bias across studies	15	Specify any assessment of risk of bias that may affect the cumulative evidence (e.g., publication bias, selective reporting within studies).<br>*-There was no substantial risk of bias (Shown in the manuscript).*	6
Additional analyses	16	Describe methods of additional analyses (e.g., sensitivity or subgroup analyses, meta-regression), if done, indicating which were pre-specified.<br>*-No sub-group analysis was performed.*	9-12
**RESULTS**			
Study selection	17	Give numbers of studies screened, assessed for eligibility, and included in the review, with reasons for exclusions at each stage, ideally with a flow diagram.<br>*-A PRISM flow chart was drawn.*	Fig 1, page 6
Study characteristics	18	For each study, present characteristics for which data were extracted (e.g., study size, PICOS, follow-up period) and provide the citations.<br>*-See Table 1.*	Done. Table 1, page 18-19
Risk of bias within studies	19	Present data on risk of bias of each study and, if available, any outcome level assessment (see item 12).<br>*-Performed fully based on Cochrane Handbook for Systematic Reviews of Interventions version 5.1.0 and presented in Supplement 4.*	Done.<br>Page 20
Results of individual studies	20	For all outcomes considered (benefits or harms), present, for each study: (a) simple summary data for each intervention group (b) effect estimates and confidence intervals, ideally with a forest plot.<br>*-Reported as forest plot as well as table including additional analyses.*	Done.<br>See Fig 3.
Synthesis of results	21	Present results of each meta-analysis done, including confidence intervals and measures of consistency.<br>*-Related table and figure were produced.*	pages 5-8, Fig 3.

Risk of bias across studies	22	Present results of any assessment of risk of bias across studies (see Item 15).<br>*-Detailed publication analyses have been performed.*	Page 14, Supp 5.
Additional analysis	23	Give results of additional analyses, if done (e.g., sensitivity or subgroup analyses, meta-regression [see Item 16]).<br>*- No subgroup analysis was performed.*	None
**DISCUSSION**			
Summary of evidence	24	Summarize the main findings including the strength of evidence for each main outcome; consider their relevance to key groups (e.g., healthcare providers, users, and policy makers).<br>*-The strength of calculations was discussed and relevance to psychiatric disorders was underlined.*	pages 12-15
Limitations	25	Discuss limitations at study and outcome level (e.g., risk of bias), and at review-level (e.g., incomplete retrieval of identified research, reporting bias).<br>*-The most important shortcomings were missing sample characteristics*	page 16
Conclusions	26	Provide a general interpretation of the results in the context of other evidence, and implications for future research.<br>*-Future research must report the information about sample characteristics in details. And, more studies are needed.*	page 15-17
**FUNDING**			
Funding	27	Describe sources of funding for the systematic review and other support (e.g., supply of data); role of funders for the systematic review.<br>*-Answer: There was no funding.*	page 1

From: Moher, D., Liberati, A., Tetzlaff, J., & Altman, D. G. (2009). Preferred reporting items for systematic reviews and meta-analyses: the PRISMA statement. *Journal of Clinical Epidemiology*, *62*(10), 1006–1012. https://doi.org/10.1016/j.jclinepi.2009.06.005

For more information, visit: **www.prisma-statement.org**.

Table S2. Comparisons of direct and indirect evidence

Random effects model:

comparison	k	prop	nma	direct	indir	Diff	z	p-value
AN:HC	36	0.93	0.70	0.77	-0.22	0.99	1.92	0.0552
Obesity:HC	15	0.82	-0.85	-1.08	0.18	-1.26	-2.56	0.0106
AN:Obesity	8	0.50	1.55	1.27	1.83	-0.56	-1.36	0.1750
BN:HC	5	0.81	0.36	0.33	0.49	-0.17	-0.20	0.8417
Obesity:Thin	3	0.59	-1.32	-1.13	-1.59	0.46	0.62	0.5364
BED:HC	3	0.78	-0.76	-1.04	0.26	-1.30	-1.30	0.1922
AN:BN	3	0.52	0.34	0.28	0.41	-0.13	-0.20	0.8447
AN:Thin	3	0.52	0.23	-0.37	0.88	-1.25	-1.76	0.0790
BED:BN	2	0.63	-1.11	-0.99	-1.33	0.34	0.35	0.7278
Thin:HC	2	0.37	0.47	0.23	0.61	-0.39	-0.52	0.6012
BED:Obesity	1	0.31	0.10	0.03	0.13	-0.10	-0.11	0.9150
AN:BED	1	0.27	1.46	-0.28	2.10	-2.38	-2.50	0.0124
BN:Obesity	1	0.18	1.21	0.75	1.31	-0.56	-0.58	0.5627
BN:Thin	1	0.26	-0.11	-1.11	0.25	-1.36	-1.28	0.1998
BED:Thin	0	0	-1.23	.	-1.23	.	.	.

Legend:

- comparison - Treatment comparison
- k - Number of studies providing direct evidence
- prop - Direct evidence proportion
- nma - Estimated treatment effect (SMD) in network meta-analysis
- direct - Estimated treatment effect (SMD) derived from direct evidence
- indir - Estimated treatment effect (SMD) derived from indirect evidence
- Diff - Difference between direct and indirect treatment estimates
- z - z-value of test for disagreement (direct versus indirect)
- p-value - p-value of test for disagreement (direct versus indirect)

Figure S1. Netheat plot

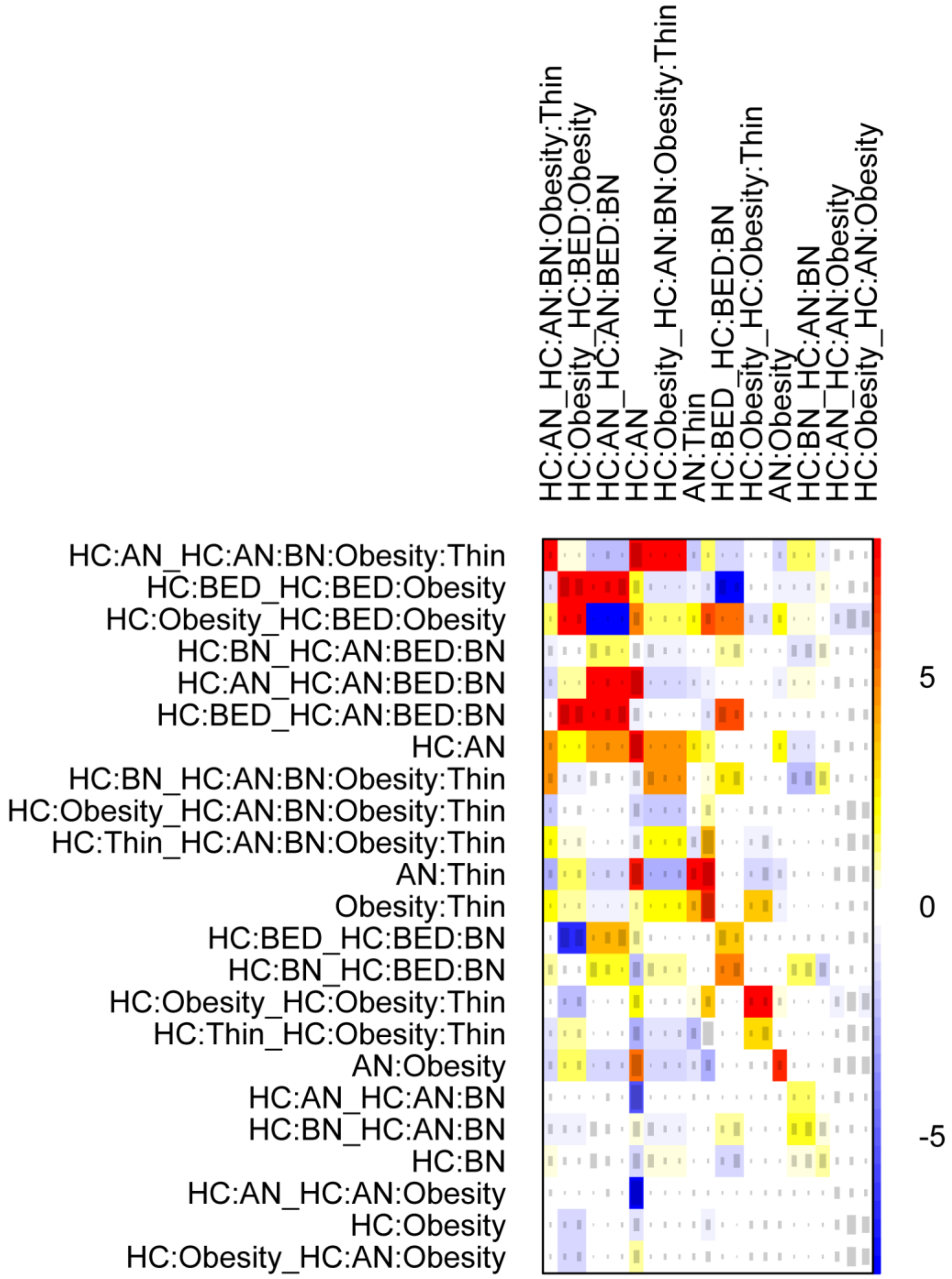

Figure S2. Risk of bias assessment

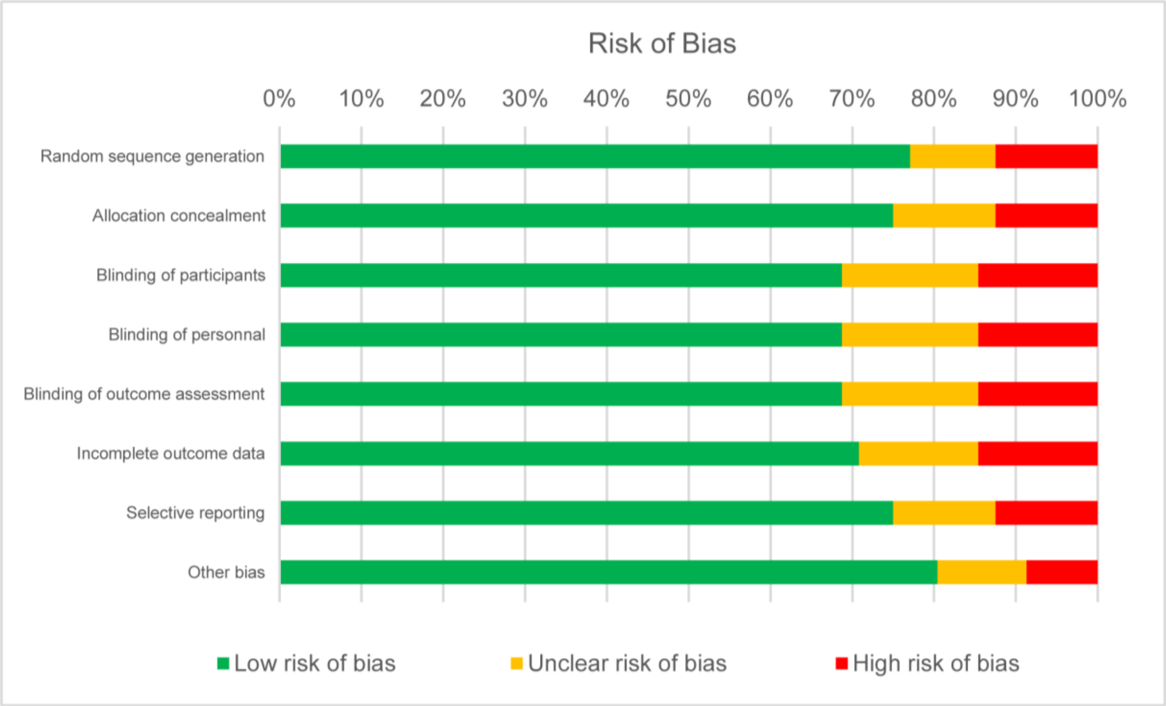

Figure S3. Funnel plot for publication bias

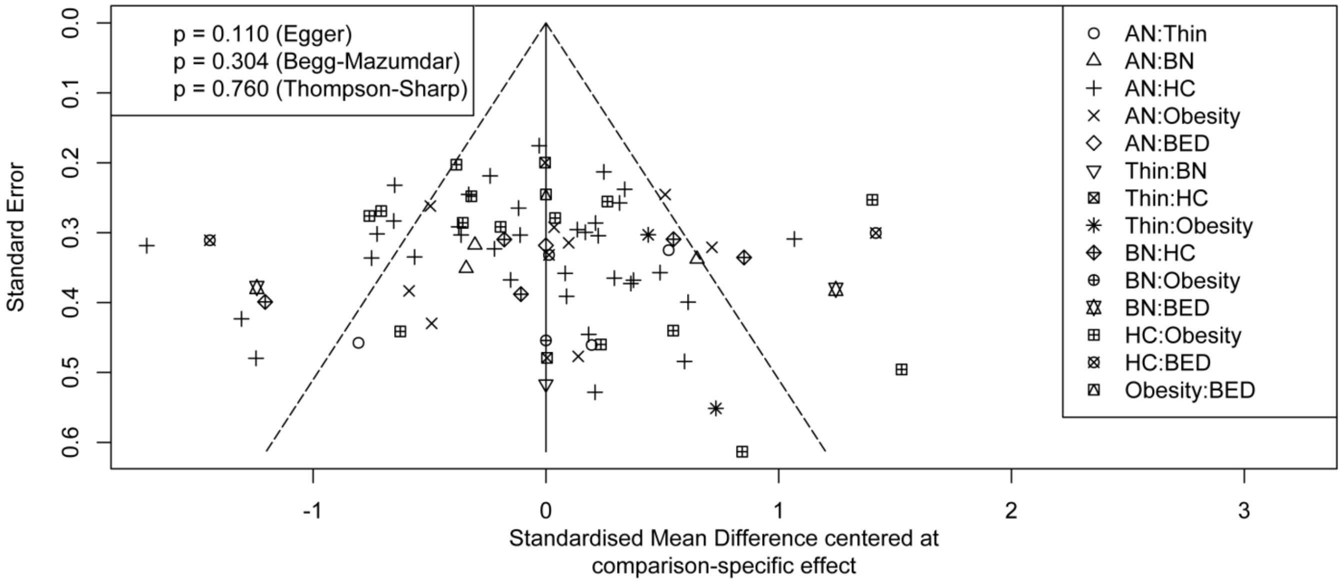

Table S4. Raw mean adiponectin levels by diagnosis

	N	k	Mean	95%-CI	Q	I^2
AN	1265	39	15.8858	[15.5827; 16.1890]	6013.03	99.4%
Obesity	867	17	0.5458	[0.5289; 0.5628]	3967.92	99.6%
HC	1572	46	0.9323	[0.8983; 0.9664]	8733.71	99.5%
BED	57	3	1.2483	[0.9500; 1.5466]	121.73	98.4%
BN	75	5	12.9945	[11.9941; 13.9950]	24.52	83.7%
C.Thin	426	5	.5177	[7.2286; 7.8068]	2476.41	99.8%
